# *Klebsiella pneumoniae* Liver Abscess in a Filipino Patient With Previously Undiagnosed Diabetes: Successful Conservative Management

**DOI:** 10.14740/jmc5377

**Published:** 2026-09-04

**Authors:** Luis Paz y Mino, Manuel Calvopina, Julia Saa, Pablo Echeverria

**Affiliations:** a One Health Research Group, Universidad de las Americas, Quito, Ecuador; b HSHS Saint Elizabeth’s Hospital, O’Fallon, IL, USA

**Keywords:** Pyogenic liver abscess, Klebsiella liver abscess, *Klebsiella pneumoniae*

## Abstract

*Klebsiella pneumoniae* is a common pathogen of pyogenic liver abscess (PLA), particularly among individuals with diabetes mellitus and those of Asian ethnicity. A 32-year-old Filipino man with no prior medical history presented with fever, headache, nausea, and vomiting and was found to have new-onset diabetic ketoacidosis (DKA). Laboratory evaluation revealed leukocytosis, thrombocytopenia, elevated liver enzymes, hyperbilirubinemia and lactic acidosis. Abdominal computed tomography (CT) showed hepatosplenomegaly, variable hepatic abnormalities including < 2 cm hypodense lesions and a large ill-defined area of decreased density in the inferior right liver lobe. The patient was hospitalized for sepsis and DKA. Empiric piperacillin–tazobactam was administered for 24 h and subsequently de-escalated to ceftriaxone after blood cultures grew extended-spectrum β-lactamase–negative *Klebsiella pneumoniae*; ceftriaxone was continued for 4 weeks. Due to the number, size and inaccessible location of the liver lesions, percutaneous drainage was not feasible. After 16-week follow-up, the patient achieved complete clinical recovery and radiological resolution of hepatic lesions. This case highlights variable hepatic lesion presentations in Klebsiella liver abscess (KLA) with successful conservative management without drainage.

## Introduction

Liver abscess is the most common type of visceral abscess [1]. While pyogenic liver abscess (PLA) predominates in developed countries, amebic/amoebic liver abscess (ALA) are prevalent in tropical endemic regions [2, 3]. Historically associated with high mortality, prompt diagnosis and management of PLA have reduced mortality rates from approximately 65% to 2–12% [1, 4]. Although PLA is frequently polymicrobial, causative organisms vary geographically. *Streptococcus* spp. and *Escherichia coli* seem to continue being the historical organisms in Western countries [5–7], whereas *Klebsiella pneumoniae* (*K. pneumoniae*) has been reported as the most common isolated pathogen in Asia, giving rise to Klebsiella liver abscess (KLA) [8, 9]. Nevertheless, KLA has increasingly been reported worldwide, raising concerns for its epidemiologic impact [6, 9, 10].

Most cases of KLA continue to occur in patients of Asian descent [10, 11]. Asian ethnicity may be a determinant for gastrointestinal colonization by some *K. pneumoniae* serotypes [10, 12]. Diabetes, however, remains one of the strongest predisposing factors for KLA [8, 13]. Some studies have described previously unrecognized diabetes diagnosed during hospitalization for PLA [14]. Nonetheless, indications for diabetes evaluation are still limited. Other risk factors associated with KLA include liver disease, gallbladder conditions, and malignancy [8, 15, 16].

Clinical and radiologic manifestations of PLA may be variable and occasionally mimic hepatic malignancy, posing diagnostic challenges [1]. Diagnosis relies primarily on imaging methods such as ultrasound or computed tomography (CT) with contrast-enhanced as the preferred imaging modality [17, 18]. KLA has more commonly been associated with the presentation of a solitary and relatively large abscess [16, 19]. Once PLA is highly suspected, aspirate cultures from the lesion should be obtained [15, 20–22]. However, some abscesses may not be amenable to percutaneous drainage, thereby limiting both diagnostic confirmation and the delivery of targeted therapy.

We report a case of KLA in a young Filipino patient with no known prior medical history who presented with diabetic ketoacidosis (DKA) and atypical hepatic lesions concerning for malignancy. The patient was successfully managed with antimicrobial therapy alone since percutaneous drainage was not technically feasible.

## Case Report

A 32-year-old Filipino male with no known prior medical history presented to the hospital with a 2-day history of headache, nausea, vomiting, and fever. On admission, laboratory evaluation revealed metabolic acidosis with a pH of 7.31 (reference range: 7.35–7.45) and anion gap of 21.3 (8–12 mmol/L) plus marked hyperglycemia of 286 mg/dL (70–99 mg/dL), consistent with DKA. Moreover, glycosylated hemoglobin was elevated to 11.5% (< 5.7%), suggesting previously undiagnosed diabetes.

**Figure 1:**
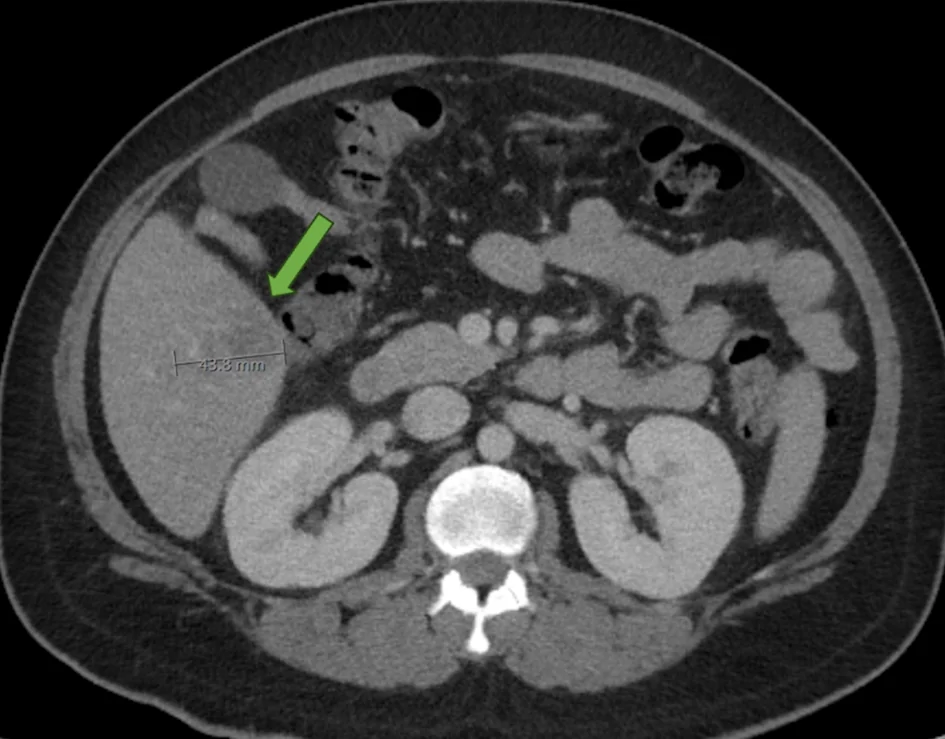
Contrast-enhanced axial abdominal CT image of the abdomen showing a 43.8-mm ill-defined hypoattenuating lesion in the inferior right hepatic lobe. The arrow identifies the larger hepatic lesion. CT: computed tomography.

Given persistent fever along with headache, a lumbar puncture was warranted during admission, and its results were unremarkable. Laboratory studies showed leukocytosis 15.8 × 10^9^/L (4.0–11.0 × 10^9^/L) with bandemia, thrombocytopenia 92 × 10^9^/L (150–400 × 10^9^/L), elevated total bilirubin 1.7 mg/dL (0.1–1.2 mg/dL) and transaminases with aspartate aminotransferase (AST) of 171 U/L and alanine aminotransferase (ALT) of 124 U/L (AST 10–40 U/L; ALT 7–56 U/L), lactic acidosis 2.2 mmol/L (0.5–2.0 mmol/L) and normal urine analysis. Chest CT reported a small left pleural effusion. CT of the abdomen demonstrated hepatosplenomegaly and multiple hypodense lesions within the liver measuring less than 2 cm, and a large ill-defined area of decreased density in the inferior right liver lobe with slight/non-substantial enhancement changes, which raised concern for underlying malignancy (Fig. 1).

On hospital day 1, the patient was admitted to the intensive care unit for management of severe sepsis with shock and DKA. Empiric antibiotic therapy with piperacillin–tazobactam was initiated due to concern for possible liver infection noted on imaging. Within 24 h of admission, blood cultures grew *K. pneumoniae* (extended-spectrum beta-lactamase negative), prompting de-escalation of antimicrobial therapy to intravenous ceftriaxone. Extensive infectious workups, including stool cultures, Shiga toxin, *Cryptosporidium* antigen, parasite ova, amoebic serology, hepatitis B and C tests were performed, and all results were negative.

**Figure 2:**
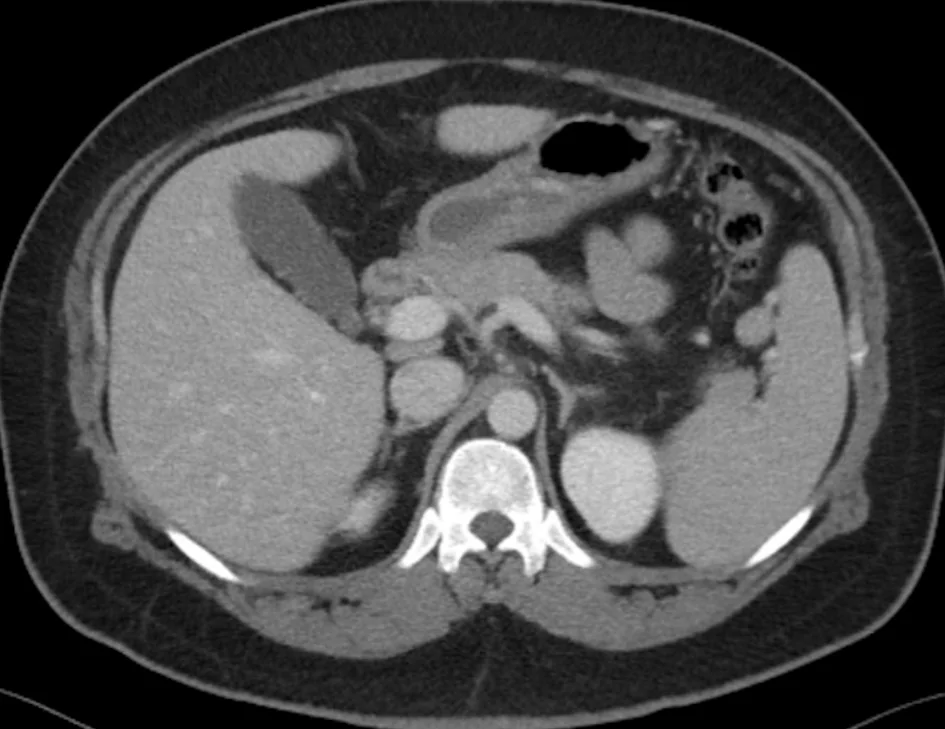
Follow-up contrast-enhanced axial CT image of the abdomen obtained 16 weeks after admission, showing complete radiological resolution with no residual hepatic lesions. CT: computed tomography.

Radiology specialists argued that hepatic lesions were deemed not amenable for percutaneous drainage given their multiplicity, small size and location. At that time, no other symptoms were reported, and ocular examination was unremarkable. Therefore, the patient was managed conservatively with prolonged antimicrobial therapy and favorable clinical response. On hospital day 3, the DKA resolved, and he was transferred from the intensive care unit. Subsequently, he was discharged home on hospital day 8 with outpatient intravenous ceftriaxone. At week 3, repeat CT imaging showed minimal change in hepatic lesions. At week 4, he completed a total 4-week course of intravenous ceftriaxone. Finally, follow-up CT imaging obtained 16 weeks after admission as part of primary care monitoring demonstrated complete resolution of all liver lesions (Fig. 2). The patient remained asymptomatic with no clinical evidence of recurrence. Furthermore, basal insulin was prescribed for type 2 diabetes mellitus and adequate glycemic control was maintained with metformin alone 3 months after discharge. The patient provided verbal consent for academic publication of this case.

## Discussion

The present case illustrates several remarkable features of PLA caused by *K. pneumoniae*. First of all, it highlights the emergence of *K. pneumoniae* as a pathogen causing PLA in the United States (USA). Secondly, it demonstrates the strong association between KLA and previously undiagnosed diabetes mellitus, including severe metabolic presentations such as DKA. Lastly, it shows that selected patients with multiple, small non-loculated abscesses not amenable to percutaneous drainage may achieve complete clinical and radiological resolution with antimicrobial therapy and careful monitoring. Our patient was originally from the Philippines, a region where KLA is well recognized, supporting previous observations that ethnicity and geographic background may influence gastrointestinal colonization with *K. pneumoniae* [10, 12].

Furthermore, this KLA case exemplifies the concomitant diagnosis of type 2 diabetes mellitus presenting as DKA. Diabetes is considered one of the strongest risk factors for KLA and has consistently been associated with increased susceptibility to invasive infection [8, 11, 13, 19]. Hyperglycemia may impair neutrophil chemotaxis, phagocytosis, and intracellular killing, facilitating dissemination of encapsulated organisms such as *K. pneumoniae* [8]. Reports have described patients in whom diabetes was first recognized during hospitalization for liver abscess [14], like our patient who had no previous diagnosis of diabetes. A glycated hemoglobin (HbA1c) of 11.5%, marked hyperglycemia of 286 mg/dL, and DKA led to a new diagnosis of type 2 diabetes mellitus. This previously unrecognized diabetes may have increased the patient’s susceptibility to *K. pneumoniae* [23]. Therefore, screening for diabetes should be considered in patients presenting with KLA.

KLA can occasionally mimic hepatic neoplasms, particularly when lesions appear infiltrative, irregular or when extensive necrosis is present on imaging studies [1, 24–26]. Liver malignancy suspicion may lead to delays in KLA recognition or unnecessary invasive procedures. Further evaluation using supportive advanced imaging could narrow the differential diagnosis, with features favoring hepatic abscess including a layered wall appearance with persistent rim enhancement, transient segmental enhancement, apparent diffusion coefficient (ADC) mapping. When the diagnosis remains uncertain, biopsy may provide definitive confirmation [1, 27, 28]. However, the hepatic lesions in this case showed only minimal enhancement, and ADC mapping—which is not routinely used or standardized for diagnosing PLA—was not obtained. In endemic regions and patients with compatible epidemiological exposures, ALA should also be included in the differential diagnosis [2, 3]. In our patient ALA diagnosis was excluded given the negative serology result. Risk factors for hepatic neoplasm were limited, and positive blood cultures for *K. pneumoniae*, together with clinical improvement after targeted antibacterial therapy, strongly supported the diagnosis of KLA.

Finally, prompt management is crucial, particularly given increasing rates of antibiotics resistance and higher risk of complications such as *K. pneumoniae* invasive liver abscess syndrome, cryptogenic KLA, and endogenous endophthalmitis [10, 11, 13, 19, 23]. In this case, cerebrospinal fluid analysis was normal, as well as ophthalmologic and chest examinations; hence no other metastatic foci of infection were identified. Nonetheless, clinicians should maintain a high index of suspicion for disseminated disease, particularly in diabetic patients [10, 11, 13, 19, 23]. Current management of PLA generally includes both antimicrobial therapy and abscess drainage, particularly for lesions that are large, multiloculated, or poorly responsive to antibiotics [1, 16, 18, 19]. Nevertheless, drainage is not always technically feasible, particularly in patients with multiple small lesions or lesions in difficult-to-access anatomical locations, as occurred in this case when the radiology team determined lesions not amenable for aspiration. Some reports suggest that selected patients with small abscesses may respond adequately to antibiotic therapy alone [1, 18]. While conservative antimicrobial management may be successful in selected patients, there is currently no universal guidance regarding lesion size thresholds (ranging from 3 to 5 cm of diameter) for drainage approaches [1, 18]. In addition, surgical intervention is generally reserved for cases in which percutaneous drainage fails to achieve adequate resolution [1, 18]. In this case, the patient had multiple lesions measuring less than 2 cm and one of 4 cm, all without loculation, and showed rapid clinical improvement after initiation of ceftriaxone. Complete radiological resolution after 16 weeks of antimicrobial therapy further supports the feasibility of non-interventional management in selected patients together with careful monitoring. Overall, KLA demands a multidisciplinary strategy encompassing clinical, radiological, microbiological, and even public health domains.

### Conclusions

In conclusion, this case highlights the evolving epidemiology of KLA in Western countries and underscores the importance of considering *K. pneumoniae* liver abscess in patients of Asian origin presenting with sepsis and newly diagnosed diabetes mellitus. KLA may present with variable imaging findings, and patients with multiple, small, nonloculated non-drainable lesions could be managed successfully with antimicrobial therapy alone and careful monitoring. Early recognition, prompt first-line antimicrobial therapy, evaluation for underlying diabetes, and close radiological follow-up remain essential components of management.

### Learning points

KLA continues spreading beyond Asia. Risk factors for KLA need to be assessed for differential diagnostic approaches, including Asian descent and diabetes. Atypical radiologic presentations pose diagnostic challenges, especially when mimicking malignancy. Abscesses may not be amenable to drainage when therapy relies on antibiotics and close monitoring.

## Data Availability

The data supporting the findings of this study are available from the corresponding author upon reasonable request.

## Abbreviations

PLA: pyogenic liver abscess
KLA: Klebsiella liver abscess
ALA: amebic/amoebic liver abscess
DKA: diabetic ketoacidosis
CT: computed tomography
ADC: apparent diffusion coefficient
*K. pneumoniae*: *Klebsiella pneumoniae*
